# Surface Temperature Control After Cardiac Arrest in the Emergency Department Without Feedback-Controlled Devices: An Observational Single-Centre Cohort Study of Routine Care

**DOI:** 10.3390/jcm15218411

**Published:** 2026-10-29

**Authors:** Adam Jakubowski, Daniel Sosiński, Grzegorz Henryk Łopieński, Jerzy Robert Ładny, Marzena Wojewódzka-Żelezniakowicz

**Affiliations:** 1 Department of Emergency Medicine, Medical University of Bialystok, 15-089 Bialystok, Poland; 2 Emergency Department, University Clinical Hospital in Bialystok, 15-276 Bialystok, Poland; 3 Students’ Scientific Circle, Department of Emergency Medicine, Medical University of Bialystok, 15-089 Bialystok, Poland; 4 Department of Anaesthesiology and Intensive Care with Operating Theatre, University Clinical Hospital in Bialystok, 15-276 Bialystok, Poland; 5 Helicopter Emergency Medical Service, Polish Medical Air Rescue, 01-934 Warsaw, Poland

**Keywords:** cardiac arrest, post-resuscitation care, temperature control, fever prevention, emergency department, surface cooling, observational cohort

## Abstract

**Background/Objectives:** Guidelines recommend active fever prevention after cardiac arrest, yet many emergency departments (EDs) lack feedback-controlled temperature-management devices. We describe what simple surface methods achieved in one ED. **Methods:** Observational cohort of 123 comatose adults with return of spontaneous circulation treated with routine care in a university ED (2017–2020; records to March 2019 analysed retrospectively, later documentation collected prospectively). Technique and length of the management period (12 h or 24 h) were chosen by the treating physician without a written protocol: ice packs (*n* = 51), ice packs plus a forced-air blanket at its lowest heated setting (32 °C; *n* = 45), a thin sheet only (*n* = 21), forced air alone (*n* = 4) or a circulating-water device (*n* = 2). Trajectories were compared in a mixed-effects model; the primary endpoint, revised post hoc from the original target of ≤36.0 °C, was fever (≥37.7 °C) after cessation. **Results:** Ice packs plus forced air gave lower temperatures than ice packs alone from 4 h (adjusted difference −0.68 °C, 95% CI −0.95 to −0.41) to 24 h (−0.44 °C), but overshot below 35.0 °C more often (34/45 vs. 9/51). Fever after cessation was more frequent after physician-selected 12 h than 24 h periods (23/33 vs. 6/90) and in the thin-sheet group (20/21). All 21 thin-sheet patients died; baseline differences suggest allocation by prognosis, and mortality was not analysed causally. **Conclusions:** Ice packs with or without forced air kept most patients below the fever threshold for up to 36 h; the combination cooled more, which did not mean better control. Safety could not be assessed, and randomised evaluation is needed.

## 1. Introduction

Sudden cardiac arrest is the third leading cause of death in Europe [1]. After return of spontaneous circulation (ROSC), hypoxic–ischaemic brain injury determines survival and neurological outcome, and fever aggravates this injury: each 1 °C above 37 °C has been associated with a more than twofold increase in the odds of an unfavourable neurological outcome [2]. The 2021 European Resuscitation Council–European Society of Intensive Care Medicine (ERC–ESICM) guidelines therefore recommended continuous temperature monitoring and active prevention of fever (>37.7 °C) for at least 72 h after ROSC [3], and the 2025 ERC–ESICM post-resuscitation care guidelines retain active fever prevention as the core of temperature control [4,5]. During the period covered by the present study (2017–2020), the applicable 2015 ERC–ESICM guidelines recommended maintaining a constant target temperature between 32 °C and 36 °C [6], and a target of 36 °C was in use in many European units after the Targeted Temperature Management (TTM) trial [7].

The evidence base for temperature control derives almost entirely from intensive care units equipped with feedback-controlled surface or intravascular devices [7,8,9]. In many healthcare systems, however, post-resuscitation care begins—and may continue for many hours—in the emergency department (ED), where such devices are often unavailable. Whether, and how reliably, simple surface methods can keep patients within a temperature target and below the fever threshold in this setting is not well described; the ongoing TEMP-CARE trial is a large randomised comparison of fever management with and without a temperature-control device [10]. Observational descriptions of what simple methods actually achieve—including how often they overshoot—may help to frame such trials and to inform departments that have no device.

We therefore analysed the routine documentation of comatose patients treated after cardiac arrest in a single ED over four years, in which the choice of surface cooling technique and of its duration was left to the treating physician. Our aims were (i) to describe the core-temperature trajectories, the frequency of overshoot and the occurrence of fever after cessation of temperature control with each technique, and (ii) to quantify the difference in temperature between the two techniques used most often—ice packs alone and ice packs combined with a forced-air blanket. Thirty-day mortality was examined as an exploratory outcome only.

## 2. Materials and Methods

### 2.1. Study Design and Setting

This was an observational cohort study based on the medical documentation of routine care in the Emergency Department of the University Clinical Hospital in Bialystok, Poland, for patients treated between 1 January 2017 and 31 December 2020. For patients treated from 1 January 2017 to 27 March 2019 the study variables were extracted retrospectively from the medical records; from 28 March 2019, the first day of the prospective part of the first author’s doctoral project approved by the Bioethics Committee, to 31 December 2020 the same variables were recorded prospectively from the documentation as care proceeded. In neither period did any study-related intervention take place: all treatment decisions, including the choice of the technique and duration of temperature control, were made by the treating physician as part of routine care, and no patient was enrolled in, or treated according to, a study protocol. The department is a tertiary-level ED with a resuscitation area in which post-resuscitation care, including sedation, mechanical ventilation and temperature control, is initiated and often continued for 24 h or longer before transfer to an intensive care unit. During the study period, the department had no feedback-controlled temperature-management device in routine use; a circulating-water device was available intermittently and was used in two patients. The study is reported in accordance with the Strengthening the Reporting of Observational Studies in Epidemiology (STROBE) statement [11]; the completed checklist is provided as Table S4.

### 2.2. Participants

Adult (≥18 years) patients with ROSC after in- or out-of-hospital cardiac arrest and a Glasgow Coma Scale score of ≤8 on arrival were eligible if post-resuscitation management with continuous oesophageal temperature monitoring was started in the ED, with or without active cooling, and core temperature was documented until 12 h after the end of the management period. Exclusion criteria applied in clinical practice were documented refusal of resuscitation, arrest due to active haemorrhage, terminal illness, known coagulopathy (antiplatelet therapy and thrombolysis were permitted), more than 120 min from arrest to the start of the management period, pregnancy, refractory cardiogenic shock (systolic blood pressure < 80 mmHg despite catecholamines or intra-aortic balloon counterpulsation), and suspected or confirmed intracranial haemorrhage.

Because inclusion required completion of the assigned management period plus 12 h of observation, patients who died or were transferred before that point are not represented in the cohort; the total number of patients with ROSC treated in the ED during the study period, and the number in whom temperature control was initiated but not documented to 12 h after cessation, could not be reconstructed from the available records; the 123 patients who met the above criteria form the analysed cohort (Figure S1). Patients were not selected on the basis of 30-day outcome or of the temperature achieved; the selection on survival to 12 h after cessation described above applied equally to all techniques, and the same eligibility criteria were used in the retrospective and the prospective period.

### 2.3. Temperature Control

All patients were undressed and covered with a single thin cotton sheet; mechanically ventilated patients received sedation with propofol and/or midazolam and analgesia with sufentanil or fentanyl, and a neuromuscular blocking agent was administered at the discretion of the treating physician (Table 1); its indications were not recorded (in departmental practice it was given for seizures or shivering), and shivering itself was not graded or systematically recorded. Drug doses, the use of antipyretics, the temperature of infused fluids and the ambient temperature of the resuscitation area were not recorded in the source documentation and cannot be reported. Core temperature was monitored continuously with an oesophageal probe and was transcribed to the chart at the start of the management period, i.e., the start of continuous core-temperature monitoring (T1), after 2, 4 and 8 h (T2–T4), at the end of the management period (T5; 12 h or 24 h after initiation), and 6 h and 12 h after cessation.

No written departmental protocol for temperature control existed during the study period. The choice of technique and of the duration of the management period (12 h or 24 h) was made by the treating physician according to individual practice; several physicians were involved over the four years, the reasons for individual choices were not recorded, and no criteria for interruption, re-cooling or escalation were specified. The target customarily applied in the department, not formalised in writing, was a core temperature of ≤36.0 °C at the end of the management period, consistent with the 32–36 °C range of the 2015 ERC–ESICM guidelines then in force [6], which, however, recommended at least 24 h of temperature control. Five patterns of management were identified in the documentation:

Thin sheet only (*n* = 21): no active cooling and no active warming; the single sheet was the only covering.

Ice packs (*n* = 51): commercially available ice packs wrapped in cloth to avoid direct skin contact were placed in both axillae and both groins and replaced by the nursing staff when thawed; the number of packs and the frequency of replacement were not standardised or recorded.

Ice packs plus forced air (*n* = 45): ice packs as above, combined with a forced-air blanket (Mistral-Air Plus warming unit MA1100-EU with a Mistral-Air full-body blanket; The Surgical Company International B.V., Amersfoort, The Netherlands) operated at its lowest heated setting of 32 °C. According to the manufacturer’s technical manual, the unit offers four settings (ambient-air fan with the heater off, 32 °C, 38 °C and 43 °C) and is intended for the prevention and treatment of hypothermia [12]. Air delivered at 32 °C is below normal skin temperature and is therefore expected to produce net convective heat loss rather than warming; delivered-air and skin temperatures were not measured. The magnitude of this heat loss depends on the gradient between the delivered air and the skin, on the area covered by the blanket and on the ambient conditions, none of which was standardised; moreover, the unit has no core-temperature feedback, so its output was not adjusted to the patient’s temperature, and any adjustment depended on the staff reading the oesophageal probe and removing the blanket or the ice packs. This use for cooling lies outside the labelled warming indication, although the device itself was not modified and was operated within its stated settings; it was part of routine departmental care and was not subject to separate ethical review. The ambient-air setting was not used.

Forced air alone (*n* = 4): the forced-air blanket at 32 °C without ice packs.

Circulating-water device (*n* = 2): CritiCool with a ThermoWrap garment (Belmont Medical Technologies, Billerica, MA, USA); the target setting was chosen by the treating physician and was not recorded.

Patients were classified according to the technique documented at the start of the management period; no change of technique was documented. The forced-air-alone and circulating-water groups were too small for any comparison and are presented individually in Table S1.

### 2.4. Endpoints

The original analysis plan, formulated for the first author’s doctoral dissertation before the 2021 guidelines were published, specified core temperature ≤ 36.0 °C at the end of the management period as the primary endpoint, reflecting the departmental target of that time. During peer review it was pointed out that this endpoint rewards overshoot and does not correspond to the fever-prevention paradigm of the current guidelines [3,4]. The order of endpoints was therefore revised after the data had been analysed, and this revision is post hoc. The revised primary endpoint is fever after cessation, defined as a core temperature ≥ 37.7 °C at 6 h or 12 h after cessation of temperature control; the threshold operationalises the ERC–ESICM criterion of >37.7 °C [3] and is used consistently throughout. Secondary endpoints are (i) the core-temperature trajectory from initiation to 12 h after cessation, (ii) core temperature ≤ 36.0 °C at the end of the management period (the original primary endpoint, retained as the historical target), and (iii) the change in core temperature from the end of the management period to 12 h after cessation. We no longer use the term “rebound”, which implied a pre-specified definition that did not exist.

Safety endpoints, added at the request of the reviewers, are the lowest core temperature recorded from the start to the end of the management period (lowest of the values at T1–T5), the proportion of patients with any such value <35.0 °C and <34.0 °C, the number of measurements > 36.0 °C during active management, any serum potassium <3.5 or >5.5 mmol/L and any serum sodium <135 or >145 mmol/L during the observation period, and bleeding or transfusion recorded in the complications field of the documentation. Arrhythmias, haemodynamic instability, skin injury, infection and shivering were not systematically recorded and are not reported. The exploratory outcome was 30-day all-cause mortality.

### 2.5. Statistical Analysis

Continuous variables are presented as mean ± standard deviation (SD) and, where distributions were skewed, as median (interquartile range, IQR); categorical variables as *n*/*N* (%). Because the thin-sheet group differed from the actively cooled groups in age, acidosis, arrest aetiology and assigned duration, and because all 21 of its patients died, it is not included in the primary comparisons; it enters only the sensitivity mixed-effects model (Table S2) and the exploratory mortality model A (Table S3), whose thin-sheet terms are reported for completeness and are not interpreted. The principal comparison, specified during revision before the revised analyses were run, is between the two large actively cooled groups, ice packs plus forced air versus ice packs alone.

Temperature trajectories were analysed with a linear mixed-effects model with a random intercept for each patient and fixed effects for technique, time point, their interaction, core temperature at initiation, age, arterial pH and lactate at initiation. Because the end-of-period and post-cessation measurements of the 12 h and 24 h management periods fall at different times from the start (12, 18 and 24 h versus 24, 30 and 36 h), the primary model was fitted on a common clock within the 24 h stratum (*n* = 80; measurements at 2, 4, 8, 24, 30 and 36 h after the start). The adjustment set (initial temperature, age, arterial pH and lactate) was chosen because these variables differed between the groups at the start (Section 3.1). A sensitivity model including all three main groups and both periods, with period-relative time points and the length of the period as a covariate, is reported in Table S2. Adjusted between-technique differences at each time point are presented with 95% confidence intervals (CI) derived from the model covariance. Binary endpoints are reported as risk differences (RD) and risk ratios (RR) with Wald 95% CI (Newcombe score interval where a cell was zero) and Fisher’s exact test; for the five binary endpoints compared between the two techniques (fever ≥ 37.7 °C at 6 h, at 12 h, at either time, at 24 h after the start, and ≤36.0 °C at the end of the period), *p*-values were adjusted with the Holm procedure [13] and all five are reported in Section 3.3. Outcomes after cessation are additionally presented stratified by the length of the management period, because the observation window after a 12 h period ends 24 h after the start, whereas after a 24 h period it ends at 36 h. No technique × period interaction was tested, because the 12 h stratum contains only four patients managed with ice packs plus forced air. No a priori sample-size calculation was performed; all eligible patients were included.

Thirty-day mortality was analysed only to describe its association with the recorded covariates. Because all patients managed with a thin sheet died (complete separation), logistic regression was fitted with Firth’s penalised likelihood [14,15], with 95% CI obtained from the profile penalised likelihood rather than from Wald standard errors. Two models are reported: model A with technique (ice packs as reference; ice packs plus forced air; thin sheet), length of the management period (12 h as reference), age, arterial pH (per 0.1 unit) and lactate at initiation; and model B restricted to the two actively cooled groups. The design matrix, the number of deaths per group and the number of events per parameter are reported with each model (Table S3). These estimates are not interpreted causally.

Temperature, blood-gas, age and outcome data were complete for all 123 patients; serum potassium was missing in one patient and sodium in two (both in the ice-pack group); no imputation was performed. All analyses were performed on the patient-level database compiled for the first author’s doctoral dissertation (2022), which has not been modified since; the single correction described below was applied in the analysis code, leaving the source file unchanged. The dissertation reported 30-day mortality in the thin-sheet group as 20/21 (95.2%) owing to a typographical error; the patient-level database records 21/21 deaths, and this value is used throughout. One lactate value stored as 120.0 mmol/L was verified against the laboratory record by the first author and corrected to 12.0 mmol/L in the analysis code; no other value was altered, and there were no duplicate records. Point estimates of the penalised-likelihood models were cross-checked between two independent implementations (Newton–Raphson iterations and direct maximisation of the penalised likelihood). Analyses were performed in Python 3.9.6 (Python Software Foundation, Wilmington, DE, USA) with pandas 2.3.3, SciPy 1.13.1 and statsmodels 0.14.6; the Firth model was implemented by the authors (code available on request). A two-sided α of 0.05 was used.

## 3. Results

### 3.1. Cohort and Allocation

A total of 123 patients were analysed (70 men, 56.9%; mean age 74.1 ± 14.8 years, median 79, IQR 65–86). Fifty-one were managed with ice packs, 45 with ice packs plus forced air, 21 with a thin sheet only, four with forced air alone and two with a circulating-water device. Baseline characteristics of the three main groups are shown in Table 1; the six patients in the two smallest groups are listed individually in Table S1.

Allocation was not balanced. Patients managed with a thin sheet were approximately a decade older, more acidotic, had higher lactate, were warmer at initiation and were assigned a 24 h management period in fewer than one-third of cases. Patients managed with ice packs plus forced air had the lowest lactate, were most often assigned the 24 h period and included almost all patients with an acute coronary syndrome as the recorded cause of arrest (10/45, versus 2/51 with ice packs and 0/21 with a thin sheet). These differences are consistent with technique and duration having been chosen according to the perceived prognosis, and they condition the interpretation of every comparison that follows.

### 3.2. Core-Temperature Trajectories

**Figure 1:**
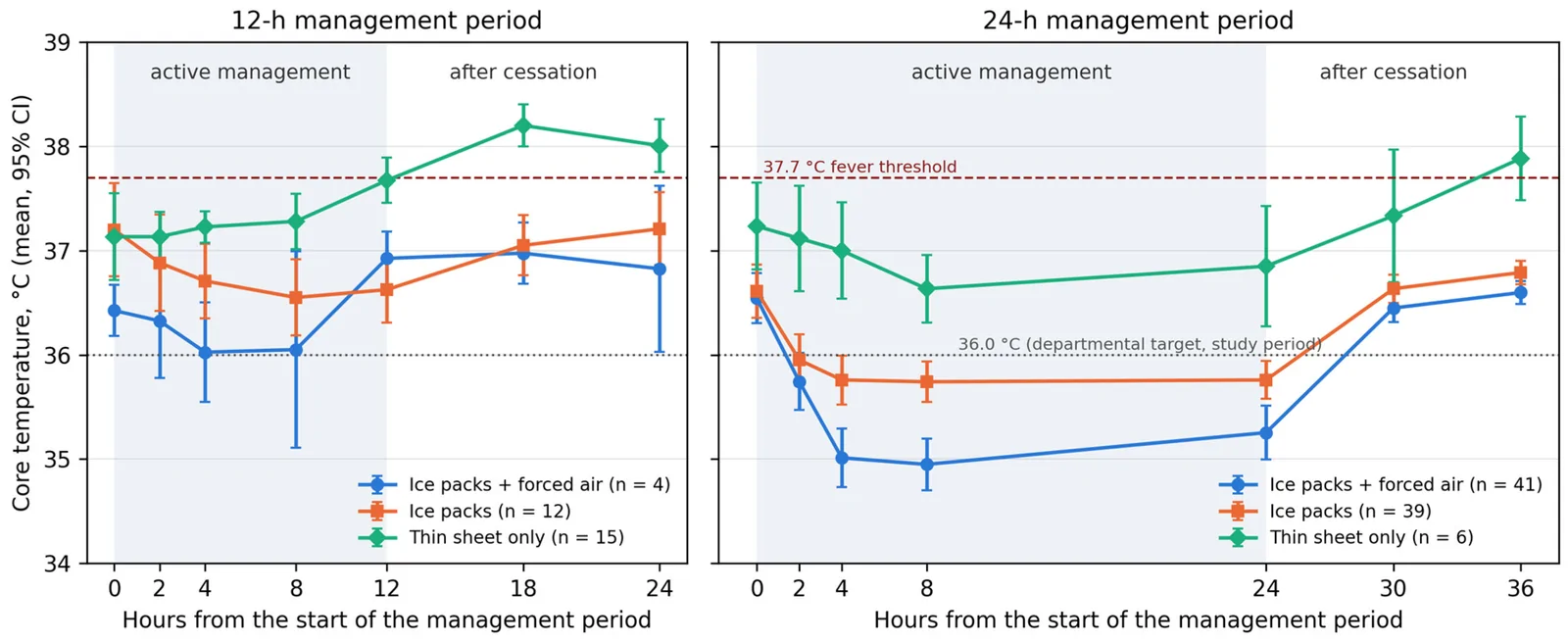
Core temperature (mean, 95% confidence interval) by technique and management period, plotted against time from the start of the management period. (**Left**): 12 h management period (ice packs + forced air *n* = 4, ice packs *n* = 12, thin sheet *n* = 15). (**Right**): 24 h management period (*n* = 41, 39 and 6, respectively). Shaded area, active management; dashed line, 37.7 °C fever threshold [3]; dotted line, 36.0 °C historical departmental target of the study period (original primary endpoint). The forced-air-alone (*n* = 4) and circulating-water (*n* = 2) patients are shown in Table 2, Tables S1 and S2.

Figure 1 shows the mean core temperature by technique on a common time axis from initiation, separately for the 12 h and the 24 h management period; the underlying values are given in Table 2 (24 h period) and Table S2 (12 h period). Under a thin sheet, core temperature did not fall: it remained between 36.6 and 37.3 °C during a 24 h period, reached a mean of 37.7 °C by the end of a 12 h period (Table S2), and exceeded the fever threshold after cessation in both strata. With ice packs, mean temperature fell by approximately 0.8 °C within 4 h and then plateaued at 35.7–35.8 °C for the remainder of the 24 h period; with ice packs plus forced air it fell to 35.0 °C at 4–8 h and was 35.3 °C at 24 h. After cessation, both actively cooled groups of the 24 h stratum rewarmed to 36.5–36.8 °C within 12 h.

In the mixed-effects model fitted on the common clock of the 24 h period (Table 3), ice packs plus forced air produced lower core temperatures than ice packs alone from 4 h onwards, by 0.68 °C at 4 h (95% CI −0.95 to −0.41), 0.72 °C at 8 h (−0.99 to −0.46) and 0.44 °C at the end of the period (−0.71 to −0.17), with no difference 6 h and 12 h after cessation. The sensitivity model including both periods and the thin-sheet group gave the same pattern (ice packs plus forced air versus ice packs: −0.71 °C at 4 h, −0.72 °C at 8 h, −0.39 °C at the end of the period; thin sheet versus ice packs: +0.39 to +0.91 °C at every time point; Table S2).

### 3.3. Fever After Cessation (Revised Primary Endpoint), Historical Target (Original Primary Endpoint) and Change After Cessation

Table 4 presents the binary endpoints by technique and management period. Fever after cessation (≥37.7 °C at 6 h or 12 h; revised primary endpoint) occurred in 20/21 patients managed with a thin sheet, 5/51 with ice packs and 2/45 with ice packs plus forced air. Its occurrence varied with the physician-selected length of the management period more than with the technique: among all patients, fever after cessation was recorded in 23/33 (69.7%) after a 12 h period and in 6/90 (6.7%) after a 24 h period, and among patients managed with ice packs with or without forced air in 6/16 (37.5%) versus 1/80 (1.3%). Within the 24 h stratum, fever after cessation occurred in 1/41 patients with ice packs plus forced air and 0/39 with ice packs alone; within the 12 h stratum, in 1/4 and 5/12.

For the principal contrast of ice packs plus forced air versus ice packs alone (all periods combined), fever after cessation occurred in 2/45 (4.4%) versus 5/51 (9.8%) patients (RD −0.05, 95% CI −0.16 to +0.05; RR 0.45, 0.09 to 2.22; Holm-adjusted *p* = 1.00), and the historical target of ≤36.0 °C at the end of the management period (the original primary endpoint) was reached in 34/45 (75.6%) versus 30/51 (58.8%) (RD +0.17, −0.02 to +0.35; RR 1.28, 0.97 to 1.71; Holm-adjusted *p* = 0.64). Within the 24 h stratum the target was reached in 34/41 (82.9%) versus 29/39 (74.4%) (RD +0.09, −0.09 to +0.26). After the 24 h period, the actively cooled groups rewarmed by a median of 0.9 °C (ice packs) and 1.2 °C (ice packs plus forced air) within 12 h of cessation, i.e., the larger rewarming followed the lower end-of-period temperature.

### 3.4. Safety

**Figure 2:**
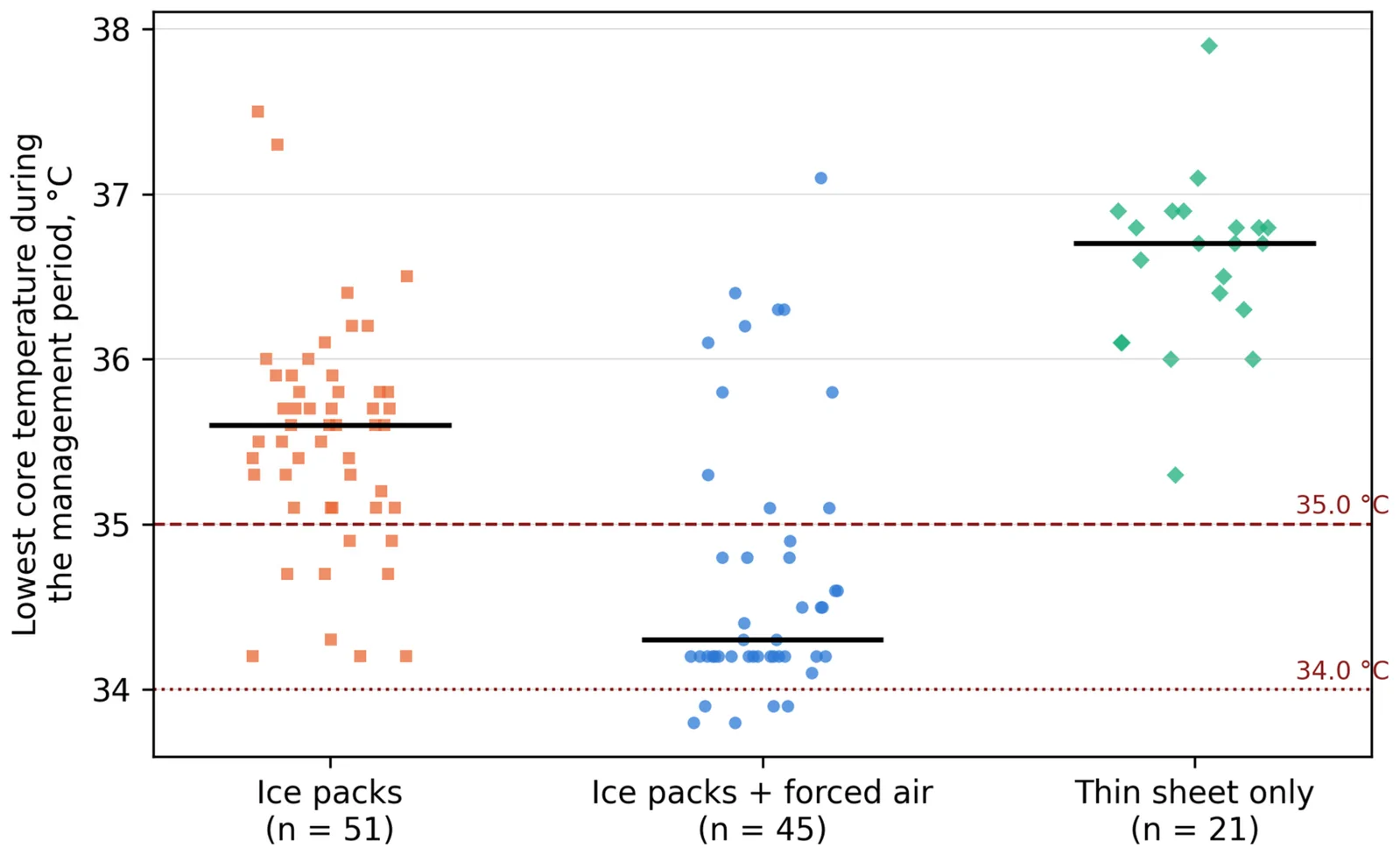
Lowest core temperature recorded from the start to the end of the management period (T1–T5) in each patient, three main groups (*n* = 117), by technique (horizontal bars, medians). Dashed line, 35.0 °C; dotted line, 34.0 °C. The six patients managed with forced air alone or a circulating-water device are listed in Table S1.

The lowest core temperature recorded during the management period and the frequency of overshoot are shown in Table 5 and Figure 2. With ice packs plus forced air, 34/45 patients (75.6%) had at least one value below 35.0 °C and 5/45 (11.1%) at least one value below 34.0 °C (lowest recorded value 33.8 °C), compared with 9/51 (17.6%) and 0/51 with ice packs alone (RD for <35.0 °C 0.58, 95% CI 0.42 to 0.74; RD for <34.0 °C 0.11, 0.02 to 0.23 by the Newcombe score method, because of the zero cell). No value below 32.0 °C was recorded in any patient. Conversely, during active management the ice-pack group had more measurements above 36.0 °C (89/204 versus 42/180). Potassium and sodium values outside the reference range were common in all groups and did not differ in a pattern attributable to technique. Bleeding or transfusion was recorded in two patients managed with ice packs and in one managed with ice packs plus forced air. Arrhythmias, haemodynamic instability, skin injury, infection and shivering were not recorded in the source documentation; the absence of a recorded complication therefore does not show that none occurred, and these data do not establish the safety of any technique. Because core temperature was transcribed at discrete time points, the lowest recorded value may also underestimate the true nadir, so the frequency and depth of overshoot reported here are minimum estimates.

### 3.5. Thirty-Day Mortality (Exploratory)

Thirty-day mortality was 21/21 with a thin sheet, 16/51 (31.4%) with ice packs, 6/45 (13.3%) with ice packs plus forced air, 0/4 with forced air alone and 2/2 with the circulating-water device. In the penalised-likelihood model including the three main groups (model A; 43 deaths, 6 parameters), the profile-likelihood CI for ice packs plus forced air versus ice packs included 1 (odds ratio 0.30, 95% CI 0.08 to 1.01), as did that for the 24 h versus the 12 h period (0.31, 0.07 to 1.32); age (1.06 per year, 1.01 to 1.12) and arterial pH (0.23 per 0.1 unit, 0.12 to 0.37) were associated with death, and the thin-sheet estimate (26.6, 2.78 to 3587) reflects complete separation. The model restricted to the two actively cooled groups (model B; 22 deaths, 5 parameters, 4.4 events per parameter) gave virtually identical point estimates with slightly wider intervals (Table S3). Given the allocation by prognosis described in Section 3.1, the absence of initial rhythm, time to ROSC and treatment-limitation decisions from the dataset, and the small number of events, these associations describe the cohort and do not estimate an effect of technique or duration.

## 4. Discussion

### 4.1. Main Findings

In an emergency department without a feedback-controlled temperature-management device, surface cooling with ice packs, with or without a forced-air blanket operated at 32 °C, lowered core temperature by 0.8–1.5 °C within 4 h and held group means between 35.0 and 35.8 °C for 24 h, although individual trajectories varied widely and the combination frequently fell below 35 °C (Section 3.4); fewer than 3% of patients managed for 24 h with either technique developed fever within 12 h of cessation. Adding the forced-air blanket produced temperatures 0.4–0.7 °C lower than ice packs alone during active management, but this difference disappeared after cessation and was achieved at the cost of frequent overshoot: three-quarters of patients fell below 35.0 °C and one in nine below 34.0 °C. By the target then in use (≤36.0 °C), the combination appeared more “effective”; by the current standard of fever prevention, the two techniques did not differ, and the combination was less precise. A lower temperature should therefore not be read as better temperature control: the current aim is to prevent fever without inducing unnecessary hypothermia, greater cooling intensity has no demonstrated clinical benefit, and the risks of the overshoot observed here could not be assessed.

Fever after cessation was more frequent after 12 h management periods and in patients without active cooling, and did not differ between the two active techniques: it occurred after 12 h periods in 70% of patients and after 24 h periods in 7%, and in 20 of 21 patients managed with a thin sheet. Because the observation window after a 12 h period closes 24 h after the start and that after a 24 h period closes at 36 h, our data describe the beginning of the 72 h fever-prevention period recommended by current guidelines [3,4]; fever appeared within hours of stopping active management in almost half of the actively cooled patients whose management stopped at 12 h. This is an association, not evidence that a longer management period prevents fever: the length of the period was chosen by the physician, the 12 h and 24 h groups differed in baseline characteristics and expected prognosis, and the observation windows were not equivalent in time from the start. Whether prolonging device-free surface cooling prevents later fever needs to be tested prospectively with standardised protocols.

Finally, the cohort demonstrates how strongly physician choice shapes observational comparisons of temperature control. Patients managed with a thin sheet were older, more acidotic and rarely assigned a 24 h period, and all of them died; patients managed with ice packs plus forced air were the least acidotic, were almost always assigned 24 h and included nearly all patients with an acute coronary syndrome. This pattern is consistent with treatment decisions made according to expected outcome. We therefore report mortality only as a description of the cohort and draw no inference about any effect of technique or duration on survival.

### 4.2. Findings in the Context of Current Guidelines and Trials

The TTM trial found no benefit of 33 °C over 36 °C [7], and the TTM2 trial subsequently showed no benefit of hypothermia at 33 °C over normothermia with early treatment of fever (≥37.8 °C) [8]; both the 2021 ERC–ESICM guidelines [3] and their 2025 revision [4,5] accordingly define temperature control primarily as active prevention of fever. In the Blood Pressure and Oxygenation Targets in Post-Resuscitation Care (BOX) trial, 36 and 72 h of device-based fever prevention did not differ in outcome [9], and a recent meta-analysis found no neurological benefit from earlier, prehospital initiation of hypothermic temperature control despite faster attainment of target temperature [16]. Whether a device is needed at all for fever prevention is the question of the ongoing TEMP-CARE trial [10]. Our data do not answer that question, but they describe what happened in one department that had no device: simple surface methods kept most patients below the fever threshold while they were applied, the combination of two methods overshot, and fever followed early cessation. In a 427-patient cohort from a centre without device-based temperature control, fever occurred in 59% of survivors and each 1 °C above 37.5 °C was associated with a 31% reduction in the odds of a good neurological outcome [17]; our cohort illustrates one circumstance in which such fever arose—short or absent active management—and the limits of the methods available to prevent it.

The overshoot observed with ice packs plus forced air is clinically relevant. Within the 32–36 °C range of the 2015 guidelines [6] values of 34–35 °C were acceptable, but under the current normothermia paradigm they represent unintended hypothermia, with its known associations with bradycardia, electrolyte shifts and coagulopathy [3]. We could not assess these consequences because arrhythmias, haemodynamic events and shivering were not recorded; potassium and sodium disturbances were frequent in every group, as expected after cardiac arrest, without a pattern attributable to technique. Without a feedback loop, the operator faces a trade-off between an insufficient and an excessive intervention, and our data quantify both sides of that trade-off for two common techniques. Whether the addition of the forced-air blanket at 32 °C is useful for fever prevention—rather than for induction of hypothermia—cannot be concluded from this study.

We did not measure costs or device availability, and we no longer characterise the techniques as “low-cost”: a circulating-water device is neither inexpensive nor universally available, and even ice packs and forced-air blankets require nursing time for replacement and monitoring. The use of a forced-air warming unit at its lowest heated setting to cool patients is outside the labelled indication of the device [12]; although the unit was operated within its specified settings and without modification, this practice should be regarded as off-label and its safety was not formally evaluated. Its cooling performance is likely to vary with blanket coverage, ambient conditions and the timing of ice-pack replacement, and without temperature feedback it depends entirely on staff responding to the core-temperature reading. Our observations are therefore not sufficient evidence to recommend the routine use of forced-air warming devices for temperature reduction.

### 4.3. Interpretation of the Thin-Sheet Group

The uniform mortality under a thin sheet should not be read as an effect of the covering. The profile of these patients—older, more acidotic, with higher lactate, with heart failure or respiratory failure as the predominant causes of arrest and with short assigned periods—is consistent with implicit decisions to limit treatment, which were not documented as such. What the temperature data do show is that, in these patients, core temperature stayed near 37 °C during the management period and exceeded the fever threshold within hours of its end; passive management did not control temperature even incidentally.

### 4.4. Practical Implications for Emergency Departments Without Temperature-Control Devices

Our data do not validate any device-free technique, and the following points are lessons from routine practice rather than recommendations. First, simple surface methods require continuous core-temperature monitoring; in our cohort both insufficient cooling and overshoot became apparent only from the oesophageal reading, and intermittent transcription of that reading may miss the nadir. Second, these methods are labour-intensive: ice packs must be replaced when thawed and both ice packs and forced air must be reduced or removed when temperature falls too far, which places the feedback function on nursing staff and should be reflected in staffing and handover. Third, the absence of a written protocol in our department meant that there were no predefined criteria for de-escalation, re-cooling or escalation; a departmental protocol specifying the target range, the temperatures at which cooling is reduced or stopped, the duration of fever prevention and its continuation after transfer to the intensive care unit is a precondition for both safe practice and future evaluation. Fourth, the high rate of fever after short management periods suggests that, where active management is started in the ED, its planned duration and the responsibility for continuing fever prevention should be explicit at handover. Whether such protocols achieve fever prevention without overshoot should be tested prospectively.

### 4.5. Limitations

The principal limitation is the mechanism of allocation. Technique and duration were chosen by individual physicians, without a protocol and without recorded criteria, and the resulting groups differ in age, acidosis, aetiology and assigned duration in a direction that indicates allocation by prognosis. This is confounding by indication of a kind that no statistical adjustment can remove, and it applies to every between-group contrast in this paper; it is least damaging for the description of what each technique achieved in terms of temperature, and prohibitive for inference about survival.

The cohort is selected. Inclusion required survival with documented temperature control to 12 h after cessation; the denominator of patients with ROSC treated in the ED during the period could not be reconstructed, and patients who died or were transferred earlier are not represented, which introduces immortal time, particularly into any contrast between the 12 h and the 24 h period. Because patients who deteriorated early are absent, the comparatively favourable temperature trajectories of the actively cooled groups describe patients who survived to the end of observation and may not apply to the broader population in whom post-resuscitation temperature control is started; for the same reason, and because of allocation by prognosis, the mortality figures are strictly descriptive. Time of death was not recorded, so time-to-event methods could not be applied. Location of arrest, initial rhythm, witnessed status, bystander resuscitation, no-flow and low-flow intervals, time to ROSC, comorbidities, vasopressor use, treatment-limitation decisions, coronary angiography and neurological outcome were not available; the absence of initial rhythm, the strongest known prognostic factor, by itself precludes any causal interpretation of the mortality models. No neurological outcome was available, and none of the temperature findings implies a benefit to patients. Drug doses, antipyretics, the temperature of infused fluids, ambient temperature, shivering, arrhythmias, haemodynamic instability and skin injury were not recorded, so the description of the interventions and of their safety is incomplete. Temperature was transcribed at seven discrete time points, so the time spent outside the target range cannot be computed and the lowest recorded value may underestimate the true nadir. The observation window ended 12 h after cessation, i.e., 24 or 36 h after the start of the management period, and therefore covers only the beginning of the 72 h period in which fever prevention is currently recommended. The primary endpoint was revised after peer review and after the data had been analysed, and the original endpoint is retained as a secondary one; both are reported. The study is single-centre, and the two smallest groups permit no inference. The cohort combines retrospectively extracted (2017–March 2019) and prospectively documented (March 2019–2020) data; the variables, their definitions and the eligibility criteria were the same in both periods, but the completeness of the complication field cannot be assumed to be equal.

## 5. Conclusions

In this observational cohort from an emergency department without a feedback-controlled temperature-management device in routine use, ice packs alone or combined with a forced-air blanket at 32 °C lowered core temperature within 4 h and kept most patients below the fever threshold during a 24 h management period and for 12 h thereafter; the combination produced lower temperatures but frequently overshot below 35 °C and offered no advantage in fever prevention, so lower temperature did not translate into better temperature control. Fever after cessation was more frequent after physician-selected 12 h management periods and in patients who received no active cooling; this is an association that requires prospective confirmation. By the revised primary endpoint (fever after cessation) the two active techniques did not differ; only by the original, historical target (≤36.0 °C) did the combination appear superior. Complications were not systematically recorded, so the absence of documented adverse events cannot be taken as evidence of safety, and allocation by prognosis precludes any conclusion about effects on survival. These descriptive data characterise the performance and the imprecision of device-free surface cooling, do not support the routine off-label use of forced-air warming devices for cooling, and may inform the design of protocols and randomised trials of fever prevention without temperature-control devices.

## Figures and Tables

**Table 1:** Baseline characteristics, sedation and outcome by management pattern (three main groups, *n* = 117).

	Thin Sheet Only (*n* = 21)	Ice Packs (*n* = 51)	Ice Packs + Forced Air (*n* = 45)
Age, years, mean ± SD	82.3 ± 10.0	73.4 ± 16.0	72.0 ± 13.7
Age, years, median (IQR)	81 (78–88)	79 (65–84)	73 (62–84)
Male, *n* (%)	12 (57.1)	27 (52.9)	27 (60.0)
Recorded cause of arrest, *n* (%)			
—Heart failure	10 (47.6)	17 (33.3)	13 (28.9)
—Respiratory failure or pulmonary embolism	9 (42.9)	20 (39.2)	12 (26.7)
—Acute coronary syndrome	0 (0.0)	2 (3.9)	10 (22.2)
—Arrhythmia or electrolyte disturbance	1 (4.8)	6 (11.8)	4 (8.9)
—Neurological	1 (4.8)	1 (2.0)	3 (6.7)
—Other (hepatic, metabolic)	0 (0.0)	5 (9.8)	3 (6.7)
Arterial pH at initiation, mean ± SD	7.15 ± 0.09	7.25 ± 0.10	7.26 ± 0.11
Lactate at initiation, mmol/L, median (IQR)	11.8 (8.7–15.0)	11.1 (7.4–15.0)	7.6 (5.2–10.1)
Core temperature at initiation, °C, mean ± SD	37.16 ± 0.74	36.75 ± 0.84	36.53 ± 0.75
24 h management period, *n* (%)	6 (28.6)	39 (76.5)	41 (91.1)
Propofol, *n* (%)	11 (52.4)	32 (62.7)	20 (44.4)
Midazolam, *n* (%)	20 (95.2)	48 (94.1)	44 (97.8)
Neuromuscular blocking agent, *n* (%)	11 (52.4)	18 (35.3)	15 (33.3)
Death within 30 days, *n* (%)	21 (100)	16 (31.4)	6 (13.3)

SD, standard deviation; IQR, interquartile range. Cause of arrest as recorded in the ED documentation (first-listed diagnosis; heart failure includes decompensated chronic heart failure; respiratory failure includes exacerbated COPD, pneumonia and pulmonary embolism; “other” comprises hepatic failure and metabolic causes); location of arrest, initial rhythm, witnessed status, bystander resuscitation, no-flow and low-flow intervals, time to ROSC, comorbidities and vasopressor use were not recorded. Between-group *p*-values are not reported because the groups were formed by physician choice and differ by design. Four patients managed with forced air alone and two with a circulating-water device are listed in Table S1.

**Table 2:** Core temperature by technique at common times from initiation, 24 h management period (°C, mean ± SD).

Hours from Initiation	0	2	4	8	24 (End)	30	36
Ice packs (*n* = 39)	36.61 ± 0.81	35.95 ± 0.78	35.76 ± 0.75	35.74 ± 0.62	35.76 ± 0.57	36.63 ± 0.43	36.79 ± 0.35
Ice packs + forced air (*n* = 41)	36.54 ± 0.78	35.74 ± 0.89	35.01 ± 0.92	34.95 ± 0.81	35.25 ± 0.84	36.45 ± 0.44	36.60 ± 0.36
Thin sheet only (*n* = 6)	37.23 ± 0.52	37.12 ± 0.63	37.00 ± 0.58	36.63 ± 0.41	36.85 ± 0.72	37.33 ± 0.80	37.88 ± 0.50
Forced air alone (*n* = 4)	36.53 ± 1.37	35.27 ± 1.28	34.80 ± 0.55	35.08 ± 0.39	34.65 ± 0.78	36.38 ± 0.22	36.52 ± 0.10

Values for the 12 h management period (hours 0, 2, 4, 8, 12, 18 and 24) are given in Table S2.

**Table 3:** Adjusted difference in core temperature, ice packs + forced air versus ice packs, 24 h management period (linear mixed-effects model, *n* = 80 patients, 480 measurements).

Hours from Initiation	Difference, °C (95% CI)
2	−0.14 (−0.41 to +0.13)
4	−0.68 (−0.95 to −0.41)
8	−0.72 (−0.99 to −0.46)
24 (end of management period)	−0.44 (−0.71 to −0.17)
30 (6 h after cessation)	−0.12 (−0.39 to +0.15)
36 (12 h after cessation)	−0.12 (−0.39 to +0.14)

Random intercept per patient (between-patient variance 0.084, residual variance 0.281); fixed effects: technique, time point, technique × time point, core temperature at initiation (+0.32 °C per °C), age (+0.011 °C per year), arterial pH (−0.54 °C per unit) and lactate (−0.002 °C per mmol/L) at initiation. CI, confidence interval.

**Table 4:** Temperature endpoints by technique and management period.

Technique	Period	*n*	Fever ≥ 37.7 °C at +6 h or +12 h After Cessation (Revised Primary Endpoint)	Core Temperature ≤ 36.0 °C at End of Period (Original Primary Endpoint; Historical Target)	Change from End of Period to +12 h, °C, Median (IQR)
Ice packs	24 h	39	0 (0.0%)	29 (74.4%)	+0.90 (+0.75 to +1.20)
Ice packs	12 h	12	5 (41.7%)	1 (8.3%)	+0.50 (−0.03 to +1.23)
Ice packs + forced air	24 h	41	1 (2.4%)	34 (82.9%)	+1.20 (+0.60 to +1.80)
Ice packs + forced air	12 h	4	1 (25.0%)	0 (0.0%)	+0.05 (−0.40 to +0.35)
Thin sheet only	24 h	6	5 (83.3%)	1 (16.7%)	+0.85 (+0.65 to +1.50)
Thin sheet only	12 h	15	15 (100%)	0 (0.0%)	+0.30 (+0.05 to +0.55)
Forced air alone	24 h	4	0 (0.0%)	4 (100%)	+1.90 (+1.62 to +2.15)
Circulating water	12 h	2	2 (100%)	0 (0.0%)	−0.80 (−0.90 to −0.70)

IQR, interquartile range. The window after cessation ends 24 h after the start of the management period for the 12 h period and 36 h for the 24 h period. Holm-adjusted *p*-values for the five binary endpoints compared between ice packs + forced air and ice packs (all periods combined): fever ≥ 37.7 °C at 6 h, 1/45 vs. 2/51 (RD −0.02, 95% CI −0.09 to +0.05), *p* = 1.00; at 12 h, 1/45 vs. 4/51 (RD −0.06, −0.14 to +0.03), *p* = 1.00; at 6 h or 12 h, 2/45 vs. 5/51 (RD −0.05, −0.16 to +0.05), *p* = 1.00; at 24 h after the start, 1/45 vs. 4/51 (RD −0.06, −0.14 to +0.03), *p* = 1.00; ≤36.0 °C at the end of the period, 34/45 vs. 30/51 (RD +0.17, −0.02 to +0.35), *p* = 0.64.

**Table 5:** Safety endpoints by technique.

	Thin Sheet Only (*n* = 21)	Ice Packs (*n* = 51)	Ice Packs + Forced Air (*n* = 45)	Forced Air Alone (*n* = 4)	Circulating Water (*n* = 2)
Lowest recorded core temperature during management, °C, median (IQR)	36.7 (36.3–36.8)	35.6 (35.1–35.8)	34.3 (34.2–34.9)	34.2 (34.1–34.3)	36.1 (35.7–36.5)
Any value < 35.0 °C, *n* (%)	0 (0.0)	9 (17.6)	34 (75.6)	4 (100)	0 (0.0)
Any value < 34.0 °C, *n* (%)	0 (0.0)	0 (0.0)	5 (11.1)	1 (25.0)	0 (0.0)
Any value < 32.0 °C, *n* (%)	0	0	0	0	0
Measurements > 36.0 °C during active management, *n*/*N*	82/84	89/204	42/180	1/16	8/8
Any potassium < 3.5 mmol/L, *n*/*N* (%)	0/21 (0.0)	11/50 (22.0)	5/45 (11.1)	0/4 (0.0)	0/2 (0.0)
Any potassium > 5.5 mmol/L, *n*/*N* (%)	5/21 (23.8)	21/50 (42.0)	17/45 (37.8)	2/4 (50.0)	1/2 (50.0)
Any sodium < 135 mmol/L, *n*/*N* (%)	14/21 (66.7)	33/49 (67.3)	36/45 (80.0)	2/4 (50.0)	1/2 (50.0)
Any sodium > 145 mmol/L, *n*/*N* (%)	4/21 (19.0)	11/49 (22.4)	9/45 (20.0)	2/4 (50.0)	0/2 (0.0)
Bleeding or transfusion recorded, *n* (%)	0 (0.0)	2 (3.9)	1 (2.2)	0 (0.0)	0 (0.0)

IQR, interquartile range. The lowest recorded core temperature is the lowest of the five transcribed values from the start to the end of the management period (T1–T5) and may underestimate the true nadir; “<35.0 °C” and “<34.0 °C” denote excessive cooling (overshoot), as distinct from the historical target (≤36.0 °C at the end of the period) and from fever after cessation (Table 4). Measurements at T2, T3, T4 and T5 (four per patient) are counted for values > 36.0 °C during active management. Electrolytes refer to any of up to five measurements per patient during the observation period; N is the number of patients with at least one measurement (potassium missing in one and sodium in two ice-pack patients). Arrhythmias, haemodynamic instability, skin injury, infection and shivering were not recorded.

## Data Availability

The patient-level data are not publicly available owing to privacy and ethical restrictions; they are available from the corresponding author on reasonable request. The analysis code is available from the corresponding author upon request.

## Supplementary Materials

The following supporting information can be downloaded at https://www.mdpi.com/article/10.3390/jcm15218411/s1, Figure S1: Patient flow; Table S1: Patients managed with forced air alone (*n* = 4) or a circulating-water device (*n* = 2), presented individually; Table S2: Core temperature for the 12-h management period, and sensitivity mixed-effects model; Table S3: Penalised-likelihood (Firth) logistic regression for 30-day mortality—exploratory; Table S4: STROBE checklist for cohort studies.
