# Supplementary material for: Surface Temperature Control After Cardiac Arrest in the Emergency Department Without Feedback-Controlled Devices: An Observational Single-Centre Cohort Study of Routine Care
Source: J Clin Med. 2026 Oct 29;15(21):8411. doi: 10.3390/jcm15218411 (PMC13763739; doi:10.3390/jcm15218411)
Supplement: Supplementary file 1 [file jcm-15-08411-s001.zip › jcm-4590561-supplementary.pdf]

Supplementary Material

Surface Temperature Control after Cardiac Arrest in the Emergency Department without Feedback-Controlled Devices: An Observational Single-Centre Cohort Study of Routine Care

Jakubowski A., Sosiński D., Łopieński G.H., Ładny J.R., Wojewódzka-Żelezniakowicz M.

Figure S1. Patient flow

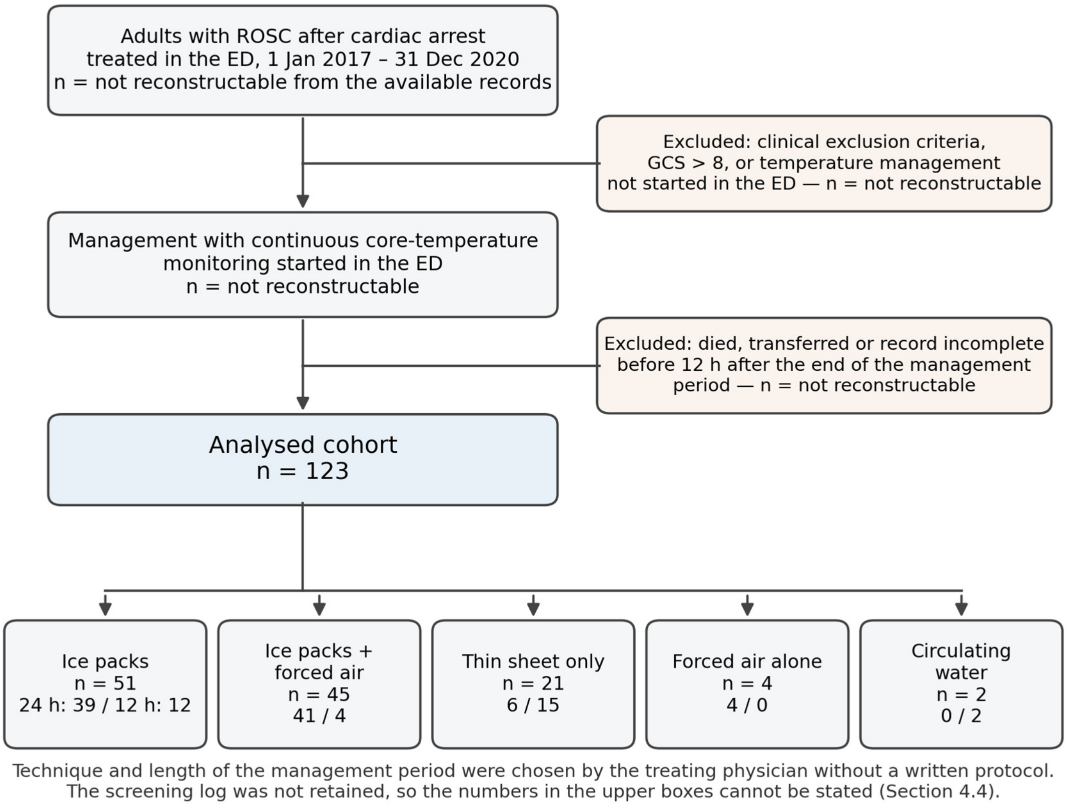

Patients with return of spontaneous circulation after cardiac arrest treated in the Emergency Department, University Clinical Hospital in Bialystok, 1 January 2017 – 31 December 2020: number not reconstructable from the available records.

→ Not meeting the clinical criteria for temperature control in the ED (documented refusal of resuscitation, arrest due to haemorrhage, terminal illness, known coagulopathy, >120 min from arrest to initiation, pregnancy, refractory cardiogenic shock, intracranial haemorrhage), Glasgow Coma Scale >8, or temperature control not initiated in the ED: number not reconstructable.

→ Temperature control initiated in the ED but documentation not available to 12 h after cessation (death, transfer or incomplete record before that point): number not reconstructable.

Analysed cohort (temperature control initiated in the ED, core temperature documented to 12 h after cessation): **n = 123.**

– Ice packs: 51 (24-h period 39, 12-h period 12).

– Ice packs + forced air: 45 (41 / 4).

– Thin sheet only: 21 (6 / 15).

– Forced air alone: 4 (4 / 0).
– Circulating-water device: 2 (0 / 2).

Patients who died or were transferred before completion of the management period plus 12 h of observation are not represented in the analysed cohort. The study database did not retain the screening log, so the numbers in the two intermediate boxes cannot be stated; this is reported as a limitation (Section 4.5).

Table S1. Patients managed with forced air alone (n = 4) or a circulating-water device (n = 2), presented individually.

Technique	Age, years	Sex	Period	T at initiation, °C	T at end of period, °C	6 h after cessation, °C	12 h after cessation, °C	Lowest T during period, °C	pH at initiation	Lactate, mmol/L	30-day death
Forced air	54	M	24 h	35.3	34.6	36.3	36.5	34.2	7.28	5.4	No
Forced air	35	M	24 h	35.6	33.7	36.6	36.6	33.7	7.32	14.1	No
Forced air	76	F	24 h	36.9	35.6	36.1	36.4	34.2	7.37	6.7	No
Forced air	72	M	24 h	38.3	34.7	36.5	36.6	34.7	7.34	12.3	No
Circulating water	84	F	12 h	35.3	37.5	37.9	36.9	35.3	7.10	10.0	Yes
Circulating water	86	M	12 h	36.9	38.5	38.9	37.5	36.9	7.11	9.1	Yes

These six patients were not included in any between-technique comparison or model. Forced air: Mistral-Air Plus MA1100-EU at the 32 °C setting with a full-body blanket. Circulating water: CritiCool with ThermoWrap garment.

Table S2. Core temperature for the 12-h management period, and sensitivity mixed-effects model.

S2a. Core temperature by technique at common times from initiation, 12-h management period (°C, mean ± SD).

Hours from initiation	0	2	4	8	12 (end)	18	24
Ice packs (n = 12)	37.20 ± 0.79	36.88 ± 0.82	36.71 ± 0.63	36.55 ± 0.64	36.62 ± 0.56	37.05 ± 0.51	37.21 ± 0.62
Ice packs + forced air (n = 4)	36.43 ± 0.25	36.33 ± 0.56	36.02 ± 0.49	36.05 ± 0.96	36.92 ± 0.26	36.98 ± 0.30	36.83 ± 0.81
Thin sheet only (n = 15)	37.13 ± 0.83	37.13 ± 0.47	37.23 ± 0.30	37.28 ± 0.53	37.67 ± 0.43	38.20 ± 0.40	38.01 ± 0.50
Circulating water (n = 2)	36.10 ± 1.13	36.75 ± 0.49	37.25 ± 0.21	37.90 ± 0.14	38.00 ± 0.71	38.40 ± 0.71	37.20 ± 0.42

S2b. Median (IQR) core temperature by technique at period-relative time points, three main groups, both periods combined (°C).

Time point	Thin sheet only (n = 21)	Ice packs (n = 51)	Ice packs + forced air (n = 45)
Initiation	37.1 (36.7–37.9)	36.7 (36.2–37.2)	36.3 (36.1–36.7)
2 h	37.1 (36.8–37.4)	36.1 (35.5–36.8)	35.6 (35.1–36.1)
4 h	37.2 (37.1–37.4)	35.9 (35.5–36.5)	34.8 (34.3–35.6)
8 h	37.2 (36.8–37.5)	35.8 (35.5–36.3)	34.8 (34.5–35.3)
End of period (12 h or 24 h)	37.6 (37.1–37.8)	36.0 (35.7–36.4)	35.3 (34.8–36.0)

6 h after cessation	38.1 (37.8–38.2)	36.8 (36.5–37.0)	36.5 (36.3–36.7)
12 h after cessation	37.7 (37.6–38.3)	36.8 (36.6–37.1)	36.6 (36.5–36.8)

S2c. Sensitivity linear mixed-effects model: three main groups, both periods, period-relative time points (n = 117 patients, 702 measurements).

Fixed effects: technique (ice packs as reference), time point (2 h, 4 h, 8 h, end of period, 6 h and 12 h after cessation), technique × time point, core temperature at initiation (+0.27 °C per °C), age (+0.008 °C per year), arterial pH at initiation (−0.85 °C per unit), lactate at initiation (−0.001 °C per mmol/L) and 24-h period (−0.62 °C vs. 12-h period). Random intercept per patient (between-patient variance 0.094, residual variance 0.263). Adjusted differences, °C (95% CI):

Time point	Ice packs + forced air vs. ice packs	Thin sheet vs. ice packs
2 h	−0.21 (−0.45 to +0.04)	+0.39 (+0.07 to +0.72)
4 h	−0.71 (−0.96 to −0.47)	+0.61 (+0.29 to +0.94)
8 h	−0.72 (−0.96 to −0.47)	+0.60 (+0.27 to +0.92)
End of period	−0.39 (−0.64 to −0.15)	+0.91 (+0.59 to +1.23)
6 h after cessation	−0.07 (−0.31 to +0.18)	+0.66 (+0.33 to +0.98)
12 h after cessation	−0.10 (−0.35 to +0.14)	+0.52 (+0.19 to +0.84)

The end-of-period and post-cessation time points fall at different times from initiation for the 12-h and 24-h periods; the primary model in the main text (Table 3) therefore uses the 24-h stratum only. Restricting this sensitivity model to the two actively cooled groups gave −0.19, −0.69, −0.70, −0.37, −0.05 and −0.08 °C at the six time points.

Table S3. Penalised-likelihood (Firth) logistic regression for 30-day mortality—exploratory.

Design. Outcome: death within 30 days. Penalty: Jeffreys invariant prior (Firth 1993); point estimates from the maximum of the penalised likelihood; 95% CI from the profile penalised likelihood (Heinze & Schemper 2002). Reference categories: technique = ice packs; management period = 12 h. Continuous covariates: age (per year), arterial pH at initiation (per 0.1 unit), lactate at initiation (per mmol/L). Patients managed with forced air alone (n = 4, 0 deaths) and with the circulating-water device (n = 2, 2 deaths) were not entered into either model.

Deaths per group: ice packs 16/51; ice packs + forced air 6/45; thin sheet 21/21 (complete separation). Deaths by length of the management period in the three modelled groups (n = 117): 12-h period 22/31; 24-h period 21/86 (all five groups: 24/33 and 21/90).

Model A—three main groups (n = 117; 43 deaths; 6 parameters excluding intercept; 7.2 events per parameter).

Design matrix columns: intercept; ice packs + forced air (0/1); thin sheet (0/1); 24-h period (0/1); age; pH × 10; lactate.

Parameter	Odds ratio	95% CI (profile penalised likelihood)
Ice packs + forced air vs. ice packs	0.30	0.08 to 1.01
Thin sheet vs. ice packs	26.6	2.78 to 3587
24-h vs. 12-h period	0.31	0.07 to 1.32
Age, per year	1.06	1.01 to 1.12
Arterial pH at initiation, per 0.1 unit	0.23	0.12 to 0.37
Lactate at initiation, per mmol/L	1.00	0.91 to 1.04

Model B—actively cooled groups only (n = 96; 22 deaths; 5 parameters; 4.4 events per parameter).

Design matrix columns: intercept; ice packs + forced air (0/1); 24-h period (0/1); age; pH × 10; lactate.

Parameter	Odds ratio	95% CI (profile penalised likelihood)
Ice packs + forced air vs. ice packs	0.30	0.08 to 1.02
24-h vs. 12-h period	0.31	0.07 to 1.32
Age, per year	1.06	1.01 to 1.12
Arterial pH at initiation, per 0.1 unit	0.23	0.09 to 0.40
Lactate at initiation, per mmol/L	1.00	0.91 to 1.04

Interpretation. Technique and period were chosen by the treating physician according to prognosis (Table 1), inclusion required survival to 12 h after cessation (immortal time favouring the 24-h period), and initial rhythm, time to ROSC and treatment-limitation decisions were not recorded. These estimates describe associations within the selected cohort and do not estimate effects of technique or duration on survival. Model B has fewer than five events per parameter.

Table S4. STROBE checklist for cohort studies.

Manuscript: Surface Temperature Control after Cardiac Arrest in the Emergency Department without Feedback-Controlled Devices: An Observational Single-Centre Cohort Study of Routine Care (Jakubowski A, Sosiński D, Łopieński GH, Ładny JR, Wojewódzka-Żelezniakowicz M). Manuscript version v08.

Study design per STROBE taxonomy: observational cohort study of routine care; records to 27 March 2019 analysed retrospectively, documentation from 28 March 2019 collected prospectively. No study-related intervention in either period. Reported using the cohort-study version of the STROBE checklist (22 items; von Elm et al., Lancet 2007; reference [11] in the manuscript). Section numbers (§) refer to the main text of v08 (1; 2.1–2.5; 3.1–3.5; 4.1–4.5; 5; Back Matter); Tables 1–5 and Figures 1–2 are in the main text; Figure S1 and Tables S1–S3 are in the Supplementary Material.

Item	Recommendation	Where reported	Note
**Title and abstract**			
1a	Study design indicated in the title or abstract.	Title; Abstract (Methods)	Title: "An Observational Single-Centre Cohort Study of Routine Care"; Abstract: "Observational cohort of 123 comatose adults … records to March 2019 were analysed retrospectively and documentation thereafter was collected prospectively, without any study intervention".
1b	Informative and balanced summary of what was done and what was found.	Abstract	Structured Background/Objectives–Methods–Results–Conclusions (259 words including section labels). States n per technique, that the primary endpoint was "re-specified after peer review", adjusted differences and risk differences with 95% CI, overshoot, and that "mortality was not analysed causally".
**Introduction**			
2	Background and rationale.	§1 (paragraphs 1–2)	"fever aggravates this injury"; "The evidence base for temperature control

			derives almost entirely from intensive care units equipped with feedback-controlled surface or intravascular devices"; EDs without devices; TEMP-CARE trial [10] as the open question.
3	Specific objectives, including any prespecified hypotheses	§1 (final paragraph); §2.4	"Our aims were (i) to describe the core-temperature trajectories, the frequency of overshoot and the occurrence of fever after cessation … and (ii) to quantify the difference in temperature between … ice packs alone and ice packs combined with a forced-air blanket"; "Thirty-day mortality was examined as an exploratory outcome only". §2.4 discloses that the original prespecified primary endpoint (≤36.0 °C at end of period) was replaced post hoc by fever after cessation.
**Methods**			
4	Key elements of study design.	§2.1; Back Matter (Institutional Review Board Statement)	"observational cohort study based on the medical documentation of routine care"; variables "extracted retrospectively" for 1 January 2017 – 27 March 2019 and "recorded prospectively from the documentation as care proceeded" from 28 March 2019; "In neither period did any study-related intervention take place".
5	Setting, locations and relevant dates (recruitment, exposure, follow-up, data collection).	§2.1; §2.3; §2.4	"Emergency Department of the University Clinical Hospital in Bialystok, Poland … between 1 January 2017 and 31 December 2020"; "tertiary-level ED with a resuscitation area"; "no feedback-controlled temperature-management device in routine use"; exposure 12 h or 24 h (§2.3); follow-up to 12 h after cessation (§2.3) and 30 days (§2.4).
6a	Eligibility criteria, sources and methods of selection, methods of follow-up.	§2.2; §2.3; §2.4	"Adult (≥18 years) patients with ROSC … Glasgow Coma Scale score of ≤8 on arrival … if temperature control was initiated in the ED and core temperature was documented until 12 h after its cessation"; nine

			exclusion criteria listed; selection on survival stated explicitly ("patients who died or were transferred before that point are not represented in the cohort"); follow-up at T1–T5, 6 h and 12 h after cessation (§2.3) and 30-day vital status (§2.4).
6b	Matched studies: matching criteria and number of exposed/unexposed.	—	Not applicable (unmatched cohort).
7	Outcomes, exposures, predictors, potential confounders and effect modifiers; diagnostic criteria.	§2.3 (exposures); §2.4 (outcomes); §2.5 (covariates)	Exposures: "Five patterns of management were identified in the documentation" and the management period (12 h or 24 h), both "made by the treating physician"; primary endpoint "fever after cessation, defined as a core temperature ≥37.7 °C at 6 h or 12 h after cessation"; secondary, safety and exploratory endpoints defined in §2.4; covariates: "core temperature at initiation, age, arterial pH and lactate at initiation" (mixed model), technique and period (Firth model). No effect modifiers were formally tested (see 12b).
8	Data sources and methods of assessment for each variable; comparability across groups.	§2.3; §2.4; §2.5; Table 5 footnote	"Core temperature was monitored continuously with an oesophageal probe and was transcribed to the chart at initiation of temperature control (T1), after 2, 4 and 8 h (T2–T4), at the end of the management period (T5 …), and 6 h and 12 h after cessation" — identical schedule for all groups; devices with manufacturer (Mistral-Air Plus MA1100-EU; CritiCool/ThermoWrap); electrolytes "any of up to five measurements per patient" (Table 5 footnote); bleeding from "the complications field of the documentation" (§2.4); mortality from the patient-level database re-verified against hospital records (§2.5). Variables not recorded in the source documentation are listed in §2.3 ("Drug doses, the use of antipyretics, the temperature of infused fluids and the

			ambient temperature … were not recorded").
9	Efforts to address potential sources of bias.	§2.2; §2.5; §4.3; §4.5; Table S3 (Interpretation)	Selection on survival disclosed (§2.2); thin-sheet group "not included in the primary comparisons"; one principal contrast; linear mixed-effects model on a common clock adjusted for baseline covariates; Holm adjustment for the five technique contrasts; Firth penalisation for complete separation with profile-likelihood CI; sensitivity model (Table S2c); confounding by indication declared not removable ("of a kind that no statistical adjustment can remove", §4.5). No quantitative bias analysis in v08 (the E-values of the previous version were withdrawn together with the causal reading of mortality).
10	How the study size was arrived at.	§2.5; §3.1; §3.5; Table S3	"No a priori sample-size calculation was performed; all eligible patients were included"; n = 123 (§3.1); events per parameter stated for the mortality models (7.2 for model A, 4.4 for model B, "fewer than five events per parameter" acknowledged in Table S3).
11	Handling of quantitative variables; groupings and why.	§2.4; §2.5; Table 3 footnote; Table 5 footnote; Table S3	Temperature analysed continuously (mixed model; mean ± SD; median (IQR)) and dichotomised at stated thresholds (≥37.7 °C "operationalises the ERC–ESICM criterion"; ≤36.0 °C "the departmental target of that time"; <35.0, <34.0, <32.0 °C; >36.0 °C at T2–T5); potassium <3.5/>5.5 and sodium <135/>145 mmol/L; age per year, pH per unit (mixed model) or per 0.1 unit (Firth), lactate per mmol/L; management period categorical (12 h vs. 24 h). Partial: the set of measurements over which "the lowest core temperature recorded during the management period" is taken is not specified (see 16b).
12a	Statistical methods, including those used to control for confounding.	§2.5	"linear mixed-effects model with a random intercept for each patient and fixed effects for technique, time point,

			their interaction, core temperature at initiation, age, arterial pH and lactate at initiation"; risk differences and risk ratios "with Wald 95% CI and Fisher's exact test"; Holm procedure [13]; "Firth's penalised likelihood [14,15], with 95% CI obtained from the profile penalised likelihood"; models A and B; software and versions; two-sided α 0.05.
12b	Methods used to examine subgroups and interactions.	§2.5; §3.3; Table 4; Table S3	Technique × time-point interaction in the mixed model; "Post-cessation outcomes are additionally presented stratified by protocol duration" (Table 4; §3.3 "Within the 24 h stratum …"); primary model restricted to the 24 h stratum; mortality model B "restricted to the two actively cooled groups". Partial: no formal test of a technique × duration interaction is reported, and the manuscript does not state that one was not performed.
12c	How missing data were addressed.	§2.5; Table 5 footnote	"Temperature, blood-gas, age and outcome data were complete for all 123 patients; serum potassium was missing in one patient and sodium in two (both in the ice-pack group); no imputation was performed"; data cleaning disclosed (one lactate value corrected after verification in the laboratory record; "no other value was altered, and there were no duplicate records"; database frozen on).
12d	How loss to follow-up was addressed.	§2.2; §2.5; §4.5	By design, patients without documentation to 12 h after cessation are "not represented in the cohort" (§2.2) and the consequences (selection on survival, immortal time) are stated in §4.5; 30-day vital status complete for all 123 (§2.5); "Time of death was not recorded, so time-to-event methods could not be applied" (§4.5).
12e	Sensitivity analyses.	§2.5; §3.2; §3.3; Table S2c; Table S3	"A sensitivity model including all three main groups and both protocols,

			with protocol-relative time points and protocol duration as a covariate, is reported in Table S2"; its results quoted in §3.2 ("gave the same pattern"); the same model restricted to the two actively cooled groups (Table S2c note); within-stratum (24 h) contrasts for the binary endpoints (§3.3); mortality model B (Table S3).
**Results**			
13a	Numbers at each stage (potentially eligible, examined, confirmed eligible, included, completing follow-up, analysed).	§2.2; §3.1; Figure S1; Table 1; Table S1	Analysed: 123, by technique and period (Figure S1; §3.1 "Fifty-one … 45 … 21 … four … two"); all 123 completed follow-up by definition. Gap: the numbers potentially eligible, examined and confirmed eligible are "not reconstructable from the available records" (§2.2; Figure S1); the placeholder in §2.2 awaits the departmental count.
13b	Reasons for non-participation at each stage.	§2.2; Figure S1	Gap: the exclusion criteria are listed (§2.2) and named in the intermediate boxes of Figure S1, but the number excluded per reason is not available ("number not reconstructable"); reported as a limitation in §4.5 ("the denominator of patients with ROSC treated in the ED during the period could not be reconstructed").
13c	Flow diagram.	Figure S1	Provided in the Supplementary Material as a text-form flow from all patients with ROSC to the 123 analysed patients, with allocation to the five techniques and the two periods; the two upper boxes carry no counts (see 13a).
14a	Characteristics of participants (demographic, clinical, social) and information on exposures and potential confounders.	Table 1; §3.1; Table S1	Age, sex, recorded cause of arrest, arterial pH, lactate, core temperature at initiation, 24 h period, propofol, midazolam, neuromuscular blocking agent and 30-day death by technique (Table 1); the six patients of the two smallest groups listed individually (Table S1); imbalance described in §3.1 ("Allocation was not balanced"). Table 1 footnote states which Utstein

			variables were not recorded ("initial rhythm, witnessed status, bystander resuscitation and time to ROSC were not recorded").
14b	Number of participants with missing data for each variable of interest.	§2.5; Table 5 footnote; Table 1 footnote; §4.5	Reported in the text, not tabulated: complete for temperature, blood gas, age and outcome; "potassium missing in one and sodium in two ice-pack patients" (Table 5 footnote). Partial: no per-variable missingness column in Table 1; variables that were not collected at all (Utstein variables, drug doses, antipyretics, shivering, arrhythmias, skin injury) are listed in the Table 1 footnote, §2.3, §2.4 and §4.5 rather than as missingness counts.
14c	Summary of follow-up time.	§2.3; §2.4; §2.5; Table 4 footnote	Fixed by design: 12 h or 24 h of management plus 12 h of observation ("The post-cessation window ends 24 h after initiation for the 12 h period and 36 h after initiation for the 24 h period"); mortality at day 30. No person-time (time of death not recorded).
15	Numbers of outcome events or summary measures over time.	§3.2; §3.3; §3.4; §3.5; Tables 2, 4, 5; Figures 1, 2; Tables S1, S2a–b	Core temperature at each time point by technique and period (Table 2, Table S2a–b, Figure 1); fever after cessation n/N by technique and period (Table 4; §3.3 "23/33 (69.7%) after a 12 h period and in 6/90 (6.7%) after a 24 h period"); target attainment and change after cessation (Table 4); overshoot, electrolyte and bleeding counts (Table 5, Figure 2); deaths per group (§3.5; Table S3 "Deaths per group").
16a	Unadjusted and confounder-adjusted estimates with precision (95% CI); which confounders were adjusted for and why.	Table 2, Table S2a (unadjusted means); Table 3, Table S2c (adjusted differences, 95% CI); §3.3, §3.4 (unadjusted RD and RR, 95% CI); §3.5, Table S3 (adjusted odds ratios, profile-likelihood CI)	Adjusted covariates listed in the Table 3 footnote and in Table S3 ("Design matrix columns: intercept; ice packs + forced air (0/1); thin sheet (0/1); 24 h period (0/1); age; pH × 10; lactate"). Why: the baseline imbalances in age, acidosis, lactate, initial temperature and assigned period (§2.5 first paragraph; §3.1). Partial: the rationale for the mixed-model covariate set is given through §3.1 rather than

			stated as such in §2.5.
16b	Category boundaries when continuous variables were categorised.	§2.4; §2.5; Table 5 footnote; Table S3	≥37.7 °C (fever), ≤36.0 °C (historical target), <35.0 °C, <34.0 °C and <32.0 °C (overshoot), >36.0 °C "at T2, T3, T4 and T5 (four per patient)" (Table 5 footnote), potassium <3.5/>5.5 and sodium <135/>145 mmol/L; pH "per 0.1 unit" (Table S3). Partial: §2.4 does not state which recorded values (T1–T5 only, or including the initiation value and the post-cessation values) enter "the lowest core temperature recorded during the management period" and "any value <35.0 °C and <34.0 °C".
16c	Absolute risk alongside relative risk, where relevant.	§3.3; §3.4; Table 4; Table 5	n/N (%) for every binary endpoint; risk differences reported alongside risk ratios ("RD −0.05, 95% CI −0.16 to +0.05; RR 0.45, 0.09 to 2.22"; "RD for <35.0 °C 0.58, 95% CI 0.42 to 0.74").
17	Other analyses (subgroups, interactions, sensitivity).	§3.3 (strata by period); §3.4 (safety); §3.5 (mortality); Table S2c; Table S3	All labelled: "Safety endpoints, added at the request of the reviewers" (§2.4); "3.5. Thirty-Day Mortality (Exploratory)"; sensitivity mixed model (Table S2c); stratification by period in Table 4.
**Discussion**			
18	Key results with reference to study objectives.	§4.1	"Main Findings": temperature achieved and overshoot with each technique (aim i and ii), fever after cessation associated with the physician-selected period and with absence of active cooling, allocation by physician choice; "By the target then in use (≤36.0 °C), the combination appeared more 'effective'; by the current standard of fever prevention, the two techniques did not differ, and the combination was less precise".
19	Limitations, sources of bias or imprecision, direction and magnitude of potential bias.	§4.5; §4.3; Table S3 (Interpretation)	Confounding by indication ("no statistical adjustment can remove"); selection on survival and immortal time with direction stated ("particularly into any contrast between the 12 h and the 24 h period"; "immortal time favouring

			the 24 h period", Table S3); absent Utstein variables ("by itself precludes a meaningful mortality model"); discrete sampling ("the lowest recorded value may underestimate the true nadir"); observation window ending at 24 or 36 h; post hoc primary endpoint; single centre; mixed retrospective/prospective periods ("the completeness of the complication field cannot be assumed to be equal"). Partial: direction of bias is given, magnitude is not quantified.
20	Cautious overall interpretation considering objectives, limitations, multiplicity, similar studies and other evidence.	§4.1; §4.2; §4.3; §5	"We therefore report mortality only as a description of the cohort and draw no inference about any effect of technique or duration on survival"; "Whether the addition of the forced-air blanket at 32 °C is useful for fever prevention … cannot be concluded from this study"; off-label use of the warming unit acknowledged (§4.2); thin-sheet mortality "should not be read as an effect of the covering" (§4.3); conclusions limited to description (§5).
21	Generalisability (external validity).	§1; §4.2; §4.5; §5	Single ED without a device; results placed against TTM [7], TTM2 [8], BOX [9], TEMP-CARE [10] and a limited-resource cohort [17]; "our data describe the beginning of the 72 h fever-prevention period recommended by current guidelines"; "The study is single-centre, and the two smallest groups permit no inference" (§4.5); practical lessons for EDs without devices, framed as non-validated (§4.4); "may inform the design of protocols and randomised trials of fever prevention without temperature-control devices" (§5).
**Other information**			
22	Source of funding and role of the funders; for the original study on which the article is based, if applicable.	Back Matter (Funding)	"This research received no external funding. The article processing charge was funded by the Medical University of Bialystok." Partial: the role of the funder

			of the article processing charge in the study is not stated. The cohort derives from the first author's doctoral project (§2.1, §2.4, §2.5), which is identified in the text.

Items not fully satisfied

13a, 13b and 13c—screening flow. The number of patients with ROSC treated in the ED during 2017–2020, the number not meeting the clinical criteria for temperature control and the number in whom temperature control was initiated but not documented to 12 h after cessation cannot be reconstructed from the available records (§2.2; Figure S1). Figure S1 therefore names the selection steps but carries counts only for the analysed cohort (n = 123) and its allocation; the §2.2 placeholder awaits the departmental count. The consequence—selection on survival to 12 h after cessation, with immortal time—is stated in §2.2 and §4.5.

14b—missing data per variable. Reported in the text (§2.5; Table 5 footnote) rather than tabulated: temperature, blood-gas, age and outcome data complete for all 123 patients; potassium missing in one and sodium in two patients, both in the ice-pack group. Variables that were not collected at all are listed in the Table 1 footnote, §2.3, §2.4 and §4.5.

16b and 11—window for the lowest recorded temperature. §2.4 defines the safety endpoints by threshold but does not state which of the seven recorded values (T1–T5, 6 h and 12 h after cessation) enter "the lowest core temperature recorded during the management period" and "any value <35.0 °C and <34.0 °C"; the counting rule is stated only for values >36.0 °C (T2–T5, Table 5 footnote).

16a—rationale for the adjustment set. The covariates of the mixed model (core temperature at initiation, age, arterial pH, lactate) are listed in §2.5 and the Table 3 footnote; the reason for their inclusion follows from the baseline imbalances described in §3.1 rather than from an explicit statement in §2.5.

12b—interactions. Stratification by management period and the technique × time interaction are reported; no formal technique × duration interaction test is reported, and the omission is not stated.

10—sample size. No a priori power calculation; the sample is the full eligible cohort of the study period (§2.5).

12d—time to death. Not recorded, which precludes survival analysis; 30-day mortality is labelled exploratory and descriptive throughout (§2.5, §3.5, §4.1, §4.5).

19—magnitude of bias. The direction of the selection and allocation biases is stated (§4.5; Table S3); their magnitude is not quantified, and v08 contains no quantitative bias analysis.

22—role of the funder. The Funding statement names the source of the article processing charge but does not state that the funder had no role in the study.

ROSC, return of spontaneous circulation; ED, emergency department; SD, standard deviation; IQR, interquartile range; CI, confidence interval; RD, risk difference; RR, risk ratio; ERC–ESICM, European Resuscitation Council–European Society of Intensive Care Medicine; T1–T5, protocol time points (initiation, 2 h, 4 h, 8 h, end of management period).
